# Supplementary material for: The interactome of intact mitochondria by cross-linking mass spectrometry provides evidence for coexisting respiratory supercomplexes
Source: Mol Cell Proteomics. 2017 Dec 8;17(2):216–32. doi: 10.1074/mcp.RA117.000470 (PMC5795388; doi:10.1074/mcp.RA117.000470)
Supplement: Supplemental Data [file supp_RA117.000470_133922_0_supp_23978_fzffwf.zip › spectra_annotation/mito_DR_spectra_annotation/291-1-1-1-14-1.pdf]

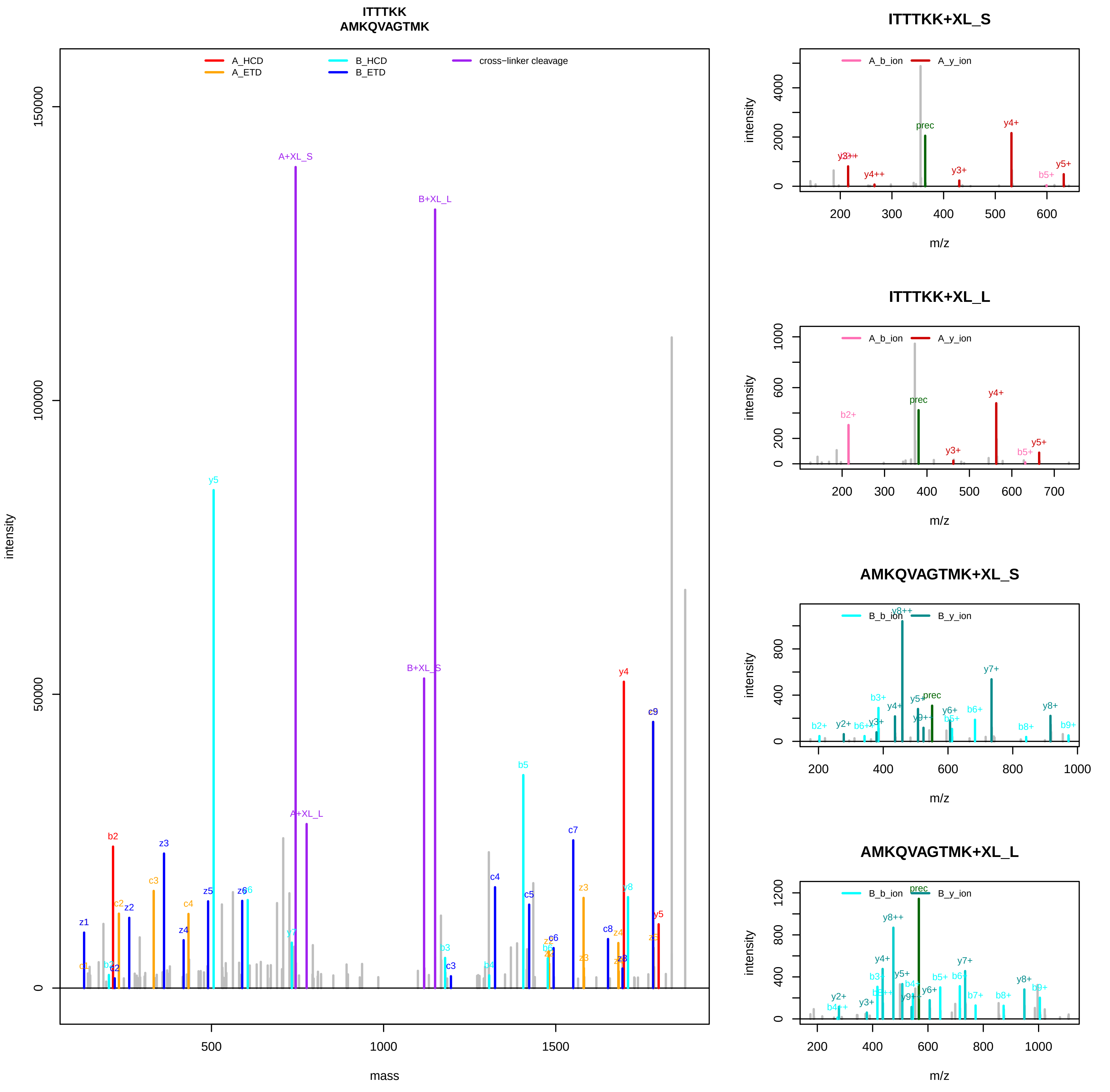

ITTTKK
AMKQVAGTMK
A_HCD
A_ETD
B_HCD
B_ETD
cross−linker cleavage
intensity
mass
0
50000
100000
150000
500
1000
1500
A+XL_S
B+XL_L
B+XL_S
A+XL_L
y5
b5
y4
c9
c7
c4
c5
c6
c8
z1
z2
z3
z4
z5
z6
c2
c3
b2
y7
y8
b3
b4
ITTTKK+XL_S
ITTTKK+XL_L
AMKQVAGTMK+XL_S
AMKQVAGTMK+XL_L
A_b_ion
A_y_ion
B_b_ion
B_y_ion
m/z
prec
y3++
y4++
y3+
y4+
b5+
y5+
b2+
y8++
y7+
y8+
b9+
b8+
b6+
y6+
y5+
b3+
y2+
